# Changes in the Bacterial Communities of Biocomposites with Different Flame Retardants

**DOI:** 10.3390/life13122306

**Published:** 2023-12-07

**Authors:** Dovilė Vasiliauskienė, Juliana Lukša, Elena Servienė, Jaunius Urbonavičius

**Affiliations:** Department of Chemistry and Bioengineering, Faculty of Fundamental Sciences, Vilnius Gediminas Technical University (VILNIUS TECH), Saulėtekio al. 11, 10223 Vilnius, Lithuania; dovile.vasiliauskiene@vilniustech.lt (D.V.); juliana.luksa@vilniustech.lt (J.L.); elena.serviene@vilniustech.lt (E.S.)

**Keywords:** biocomposites, Next Generation Sequencing, bacterial communities, flame retardants

## Abstract

In today’s world, the use of environmentally friendly materials is strongly encouraged. These materials derive from primary raw materials of plant origin, like fibrous hemp, flax, and bamboo, or recycled materials, such as textiles or residual paper, making them suitable for the growth of microorganisms. Here, we investigate changes in bacterial communities in biocomposites made of hemp shives, corn starch, and either expandable graphite or a Flovan compound as flame retardants. Using Next Generation Sequencing (NGS), we found that after 12 months of incubation at 22 °C with a relative humidity of 65%, Proteobacteria accounted for >99.7% of the microbiome in composites with either flame retardant. By contrast, in the absence of flame retardants, the abundance of Proteobacteria decreased to 32.1%, while Bacteroidetes (36.6%), Actinobacteria (8.4%), and Saccharobacteria (TM7, 14.51%) appeared. Using the increasing concentrations of either expandable graphite or a Flovan compound in an LB medium, we were able to achieve up to a 5-log reduction in the viability of *Bacillus subtilis*, *Pseudomonas aeruginosa*, representatives of the *Bacillus* and *Pseudomonas* genera, the abundance of which varied in the biocomposites tested. Our results demonstrate that flame retardants act on both Gram-positive and Gram-negative bacteria and suggest that their antimicrobial activities also have to be tested when producing new compounds.

## 1. Introduction

Global policies promote a responsible approach to consumption to minimize environmental harm by using environmentally friendly materials to reduce waste and pollution and generate sustainable economic development [1,2]. Environmentally friendly materials can be reused or can degrade naturally after their decommissioning. The creation of environmentally friendly thermal insulation materials for construction uses basic raw materials of plant origin, such as fibrous hemp, flax, bamboo, etc., as well as recycled materials, such as textile waste (cotton) and paper waste (wood). All of these plant-derived materials share a similar chemical composition, which includes cellulose, lignin, hemicellulose, pectin, and ash (inorganic substances) [3]. In regions where specific plants thrive, countries leverage the growth of these plants for various purposes. For example, in Lithuania’s climatic zone, willows are commonly cultivated as a renewable energy source for biofuel production, while fiber hemp is grown to produce ecological textiles and as a thermal insulation material. Fiber hemp is also utilized in biobased composites, which combine filler materials with starch as an organic binder and as a flame retardant [4]. Composites containing plant-derived organic matter must meet certain technological specifications, such as thermal conductivity, mechanical resistance, fire resistance [5], and, most importantly, durability. The cellulose molecules are organized in parallel (in flax, hemp, and cotton) and comprise a fiber formed by linear molecules joined via β (1→4)-glycosidic bonds. The main characteristic feature of the structure of these fibers is the presence of crystalline and amorphous regions. Amorphous sites, characterized by larger gaps between glucose molecules, are a consequence of intramolecular hydrogen bonds and Van der Waals forces being twisted and torqued. These alterations weaken their interaction, allowing water molecules to enter and consequently form hydrogen bonds with cellulose macromolecules. The enhanced hydrophilicity of the cellulose macromolecule produces a favorable environment for the growth of microorganisms. 

One of the most popular flame retardants used in biobased composites is expandable graphite, which is obtained from natural graphite with an incorporated blowing agent positioned between the layers. When exposed to flames, the intercalation compounds decompose into gaseous products that evaporate, causing the pressure between the graphite layers to increase. As a result, the graphite layers expand and exfoliate, increasing the volume of graphite by up to 300 times and significantly reducing its density. This expansion also leads to a tenfold increase in surface area. The expandable graphite forms an insulating carbon layer that acts as a protective barrier against heat and flame, while the release of water and gaseous substances also helps to quench the flame’s combustion [6,7]. The particle size of expandable graphite has a significant effect on flame retardancy. Additionally, even when not expanded, these materials are considered as flame-retardant additives in multicomponent systems [8]. The expandable graphite is also used as a sorbent of heavy oils, in supporting various catalysts, as an antimicrobial agent, and as a raw material for graphene production [9]. Graphene materials are known for their antimicrobial properties [10]. Most allotropic forms of carbon, such as graphene oxides, carbon nanotubes, and fullerenes, have been shown as moderately hazardous to the majority of living cells—both eukaryotic and, particularly, prokaryotic [11,12]. However, it has also been observed that these materials can promote the proliferation of electroactive bacteria [13].

Other types of flame retardants used for biobased composites are methylphosphonate-, dicyanamide-, or triethanolamine-based [14,15]. These materials have demonstrated potential antimicrobial properties. Notably, phosphonates have been identified as inhibitors of specific biosynthesis pathways and are primarily degraded by certain prokaryotic microorganisms [16]. Usually, the dicyanamides are used as catalysts in the synthesis of flame retardants [17]. Furthermore, under dicyandiamide catalysis, the phosphonic acid group forms phosphonic anhydride, which then reacts with hydroxyl groups on the cellulose molecule to generate numerous P-O-C covalent bonds by directly grafting onto the cellulose fiber to produce phosphate cellulose, imparting efficient flame retardancy and durability [18]. Triethanolamine is used in the production of flame retardants as a crosslinking agent [19]. It is also reported that mono-, di-, and triethanolamine inhibit the growth of a variety of microorganisms [20,21]. 

Microorganisms are ubiquitous in the biosphere, and their presence inevitably influences the surrounding environment. The effects of microorganisms on their environment can be either advantageous, harmful, or hardly detectable regarding human measures or observation. They affect traditional civil engineering materials, such as wood, concrete, and ceramics but also biobased composites made of wood, paper, or agricultural waste. Numerous studies have focused on the microbiota of larvae that decompose wood and its products or on pure cultures of fungi and bacteria [22,23,24]. In most cases, studies approach from either the perspective of physical and mechanical properties or from the microbiological viewpoint, ignoring the other approach. While it is acknowledged that plants, including hemp (*Cannabis sativa* subsp. *sativa*), serve as hosts for microbial communities [25,26], the exploration of potential shifts within the microbiota of biocomposites made from hemp shives and corn starch during exploitation remains obscure. The microbiological aspects of composites and their potential variations present an understudied domain within the existing literature. 

Hemp shives are rich in lignocellulose molecules, which are complex polymers of glucose and other sugars that can be broken down by microorganisms into simpler compounds. Consequently, they return to the biological transformation cycle, serving as a source of nutrients for plants or other organisms. It is well known that hemp shives contain significant amounts of cellulose (33–44%), a polymer of glucose, which is the main carbon source for microorganisms. In the presence of excess moisture, it penetrates the shives through the amorphous sites of the cellulose structure [27]. Likely, microorganisms will also find amorphous segments within the cellulose chain, facilitating penetration and enzymatic degradation of the polysaccharide.

In this study, our focus was on exploring the microbiota compositions within specific composites, which additionally incorporated two distinct types of flame retardants: the expandable graphite and a specialized solution containing phosphorus and nitrogen sold under the “Flovan CGN-01” trademark. We also included composites devoid of any flame retardant as control samples. By employing the Next Generation Sequencing (NGS) analysis, this research aims to demonstrate how the presence of flame retardants influences the composition of bacteria. We were able to demonstrate that control composites without flame retardants contain a plethora of bacteria, which indicates that the flame retardants used to produce composites inhibit bacterial growth and reduce the diversity of bacteria in biocomposites. We also provided evidence of the antimicrobial effect of flame retardants by analyzing the efficiency against both Gram-negative (*Pseudomonas aeruginosa*) and Gram-positive (*Bacillus subtilis*) bacteria. Our findings shed light on the inhibitory action of these flame retardants on bacterial viability and highlight their high potential as antimicrobial agents protecting biocomposites from detrimental microbial contaminations harmful to humans and negatively affecting their properties. 

## 2. Materials and Methods

### 2.1. Biocomposite Board Formation

Biocomposite boards (BcBs) were made from hemp shives (with particle sizes of 2.5 to 5 mm), and corn starch was used as a binder as described previously [28]. Flame retardants—such as expandable graphite (EG 96 M100 10 (C_24_(HSO_4_)(H_2_SO_4_)_2_), ProGraphite GmbH, Untergriesbach, Germany), or a multifunctional aqueous mixture based on the Flovan CGN-01 phosphorus and nitrogen organic compound (Huntsman, Basel, Switzerland)—were used. The board manufacturing process involved subjecting the mixture to pressing, reducing its volume to 40% of the initial volume at 0.8 MPa. Subsequently, the specimens underwent a thermal treatment involving temperature elevation (1 h to 160 °C), maintenance (6 h at 160 °C), and decrease (3 h to room temperature).

The base of BcBs-forming mixtures included hemp shives and 10% corn starch as the binder (H—composition). The G—composition featured an expandable graphite, constituting 20% of the starch mass. Additionally, the F composition incorporated a 30 g/L aqueous mixture of the Flovan flame retardant.

### 2.2. Genomic DNA Extraction from Biobased Composite Boards

The genomic DNA (gDNA) of microorganisms was isolated from BcBs after incubation over 12 months under a constant temperature of 22 °C with a relative humidity of 65% and from hemp shives which were stored at the same temperature but at 55% humidity. The 1 ± 0.05 g samples of BcBs or hemp shives were placed in 10 mL of sterile 0.9% NaCl solution and shaken at 120 rpm at a temperature of 37 °C overnight. These outwashes were centrifuged at 12,000× *g* for 20 min, and precipitates were used for subsequent analysis. Total genomic DNA (gDNA) was extracted from 250 μL of the outwashes after centrifugation of H-, G-, and F-type BcBs and hemp shives using the PureLink Microbiome DNA Purification Kit (Invitrogen, Carlsbad, CA, USA), following the manufacturer’s instructions for soil samples. Briefly, this method involved a comprehensive breakdown of various microorganisms, including robust species with dense and intricate cell walls by a combination of heat, chemical action, and mechanical disruption utilizing specialized beads followed by the removal of inhibitors by precipitation using a proprietary cleanup buffer. DNA samples were then applied to the spin column, washed, and eluted using the respective buffers. The resulting gDNA was stored at −20 °C until further analysis by NGS. Its quantity and quality were checked using agarose gel electrophoresis, and the samples later passed quality control at Macrogen Inc. (Seoul, Republic of Korea) before amplification and sequencing.

### 2.3. Next Generation Sequencing

NGS of 16S rRNA gene amplicons was performed at Macrogen Inc., using the Illumina MiSeq sequencing platform. The following oligonucleotide sequences were used for the amplification of the 16S rRNA gene, targeting the V3–V4 region from 16S rRNA primers specific to bacteria.

Forward: 5′-TCG TCG GCA GCG TCA GAT GTG TAT AAG AGA CAG CCT AC-GGGNGGC WGC AG-3′.

Reverse: 5′-GTC TCG TGG GCT CGG AGA TGT GTA TAA GAG ACA GGA CTACHVGGG TAT CTA ATC C-3′.

### 2.4. Bioinformatic Data Analysis

The sequence data generated by Macrogen Inc. were processed and analyzed using QIIME2 v2020.06 [29]. Amplicon primers were removed using Cutadapt 2.8 [30]. The DADA2 plugin was utilized for denoising, filtering, and trimming the reads, with a median quality rating threshold of 30. The RDP database (release 11) [31] was employed to classify amplicon sequence variants (ASVs) in QIIME2, with a classifier trained on the amplified region. For the dataset, the majority taxonomy with seven levels was used, which was taken to the genus level due to incomplete species-level identification.

### 2.5. Assessing the Impact of the EG and Flovan Solutions on Bacterial Growth

*Bacillus subtilis* ATCC 6633 and *Pseudomonas aeruginosa* ATCC 27853 were propagated in a Luria-Bertani (LB) medium (2% tryptone, 2% yeast extract, 1% NaCl) for 16–18 h under continuous shaking at 37 °C. To prepare the bacterial cells for the viability experiment, *B. subtilis* and *P. aeruginosa* cells grown overnight were collected by centrifugation at 8000× *g* for 5 min, washed twice with a 0.9% NaCl solution, and resuspended in 0.9% NaCl at a final concentration of around OD_600_ = 1. To investigate the effects of expandable graphite or Flovan on bacterial growth, the bacterial suspensions (300 µL) were mixed with equal volumes of either 6%, 2%, or 1% solutions of EG or FL in 0.9% NaCl. The choice of the 1% final concentration of flame retardants was based on its relevance to formed biocomposites. We also examined how lower (0.5%) and higher (3%) final concentrations of EG or FL influenced cell viability. To the control samples, 300 µL of 0.9% NaCl was added. The mixtures were incubated at 37 °C for 2 h. After incubation, serial dilutions of the bacterial suspensions were performed in a 0.9% NaCl solution, and 50 µL of each solution was spread onto an LB agar medium and incubated overnight at 37 °C. The resulting colonies were counted to determine the number of colony-forming units (CFUs) in each sample, and then the mean value of CFU/mL was calculated. All experiments were performed in triplicate. The Shapiro–Wilk test was used to verify the normal distribution. One-way analysis of variance ((ANOVA); *p* < 0.05) was performed to define statistically significant results.

## 3. Results

### 3.1. Bacterial Composition of BcBs and Hemp Shives 

**Figure 1:**
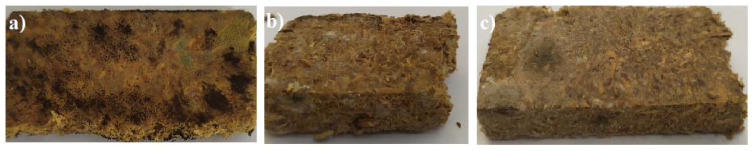
Microbial growth on the biocomposite boards: (**a**) biocomposite boards without additives (H-type); (**b**) F-type of biocomposite boards with the multifunctional aqueous mixture based on phosphorus and nitrogen organic compounds Flovan; (**c**) G-type biocomposite boards with the expandable graphite.

The colonization of microorganisms on the surface of biocomposites generates different alterations in the topography of the surface. The most noticeable effects include the presence of microorganism colonies and the penetration of the hyphae of fungi into the biocomposites, resulting in cracks and voids on the surface. As described previously [32], microbial assemblages are formed inside the cellulosic materials and/or composite boards (Figure 1). Even though after the visual evaluation one can conclude that the addition of flame retardants affects microbial growth, changes in the composition of the communities obtained cannot be determined.

To gain further insights into the impact of flame retardants on microbial communities within hemp composite materials, we conducted a comprehensive analysis of bacterial community composition. To achieve this, NGS targeting the V3–V4 region of the 16S rDNA gene was employed to determine the composition of bacterial communities in various BcBs. To establish a baseline comparison, raw hemp shives, which serve as the primary constituent of BcBs, were included as control samples (KS sample).

The BcBs compositions had three variations: with 10% corn starch as a binder (composition H); with 10% corn starch and EG (20% of the starch mass, composition G); and with 10% corn starch and a 30 g/L concentration of the Flovan flame-retardant aqueous mixture (composition F). After quality filtering and the removal of chloroplasts, a total of 194,132 high-quality reads were retained within the range of 42,038 to 59,534 reads per sample (Table 1). We utilized the DADA2 algorithm to process the Illumina sequencing data, which allowed us to assign 494 unique ASVs. The H sample, which consists of hemp shives with only corn starch as a binder, exhibited a significantly higher number of ASVs compared to other samples, with 390 unique variants. The Shannon diversity and Faith’s phylogenetic diversity indexes for this sample were 6.3 and 40.35, suggesting a high level of microbial richness and variety. The raw hemp shive (KS) sample had 52 unique ASVs, the Shannon diversity index for this sample was 4.2, and Faith’s index was 2.59, suggesting a substantially lower level of microbial diversity. This was followed by the F-type composite (37 ASVs, 3.3 Shannon, 1.45 Faith’s index) and the G-type composite (15 ASVs, 2.1 Shannon, 3.97 Faith’s index).

**Figure 2:**
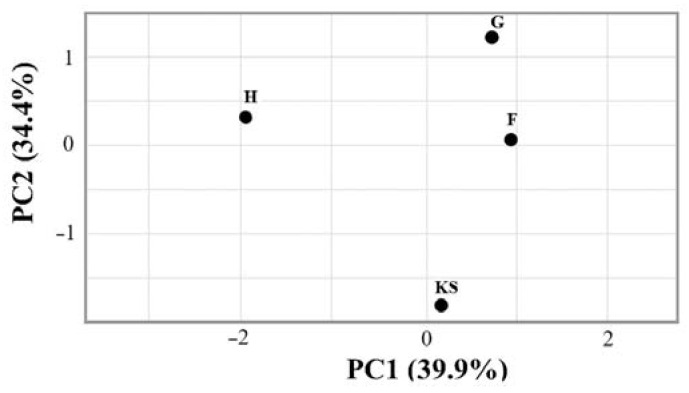
Principal coordinate analysis of microbial community structures in biocomposite samples. KS—hemp shives as a raw material for biocomposites; F, G, H—hemp-based composites, where F was with Flovan additives, G with EG, and H without additives.

The beta diversity analysis (Figure 2) revealed distinct microbial community structures among the biocomposite samples, with varying levels of dissimilarity. It can be observed that the samples with additives (F and G samples) showed relatively low dissimilarity. The KS sample exhibited a moderate level of dissimilarity with these samples. The H sample displayed a slightly higher dissimilarity compared to other samples.

**Figure 3:**
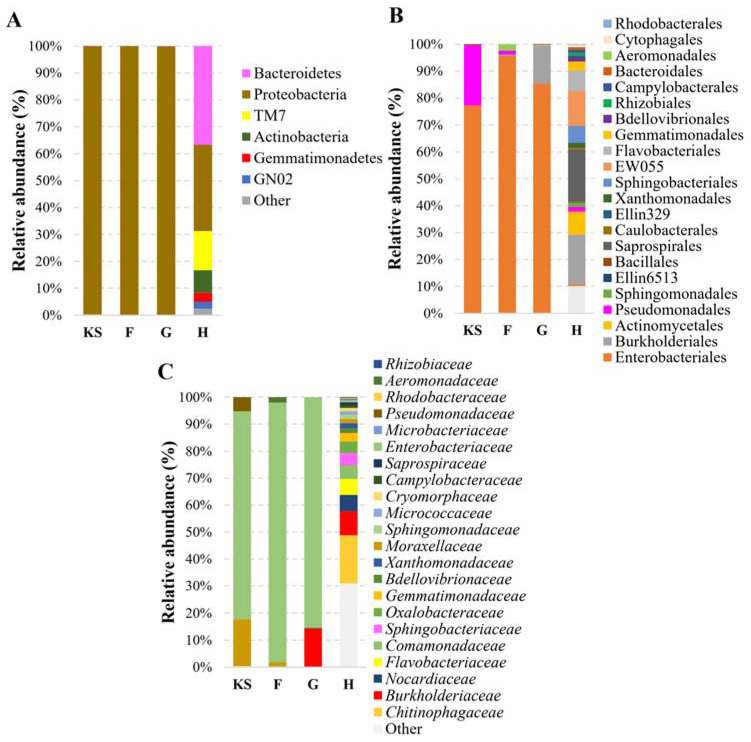
The composition of bacterial communities in different materials. KS—hemp shives as a raw material for biocomposites; F, G, H—hemp-based composites, where F is with Flovan additives, G with EG, and H is without additives. Relative bacterial abundance classified at phylum (**A**), order (**B**), and family (**C**) levels.

Figure 3 demonstrates the relative abundance of bacteria in the samples investigated. The dominant bacteria observed at the phylum level in hemp shives were identified as Proteobacteria, accounting for 99.7% of the microbial composition. 

This dominance persisted in composites with expandable graphite (G-type board, 99.7%) or Flovan (F-type board, 100%) as flame-retardant additives. In contrast, in the absence of flame retardants, the diversity of microorganisms increased: the abundance of Proteobacteria that prevail in hemp shives decreased to 32.1%, whereas Bacteroidetes (36.6%), Actinobacteria (8.4%), and Saccharobacteria (TM7) (14.51%) appeared (Table S1).

Further analysis at the order level revealed that Enterobacteriales dominated the composite samples, constituting 95.78%, 85.37%, and 77.22% of the microbial community in the F, G, and KS samples, respectively. In contrast, the H sample showed a significantly reduced abundance of Enterobacteriales, with the latter accounting for only 0.726% of the microbial community. An increased presence of Burkholderiales was observed in the G (14.19%) and H (18.47%) samples, while Actinomycetales were found mostly in the H (8.42%) sample. Interestingly, Pseudomonadales appeared in the highest abundance in the KS sample (22.43%), while the other composite samples showed significantly lower abundances (less than 2%) of this order. At the family level, the H-type composite exhibited a higher abundance of the *Chitinophagaceae* family (17.90%). Conversely, the F and G samples showed higher abundances of *Enterobacteriaceae* (95.78% and 85.37%, respectively), with relatively lower representations of other bacterial families. The KS sample, on the other hand, displayed a diverse distribution of bacterial families, including *Moraxellaceae* (17.27%) and *Enterobacteriaceae* (77.22%) (Figure 3, Table S1).

These findings suggest that the inclusion of flame-retardant additives inhibits the growth of certain types of bacteria in BcBs. It was also noticed that bacteria of the *Pseudomonas* genus were found in boards made without flame retardants (H-type). Since *P. putida* bacteria have been isolated and analyzed previously [33], it was assumed that these bacteria might be sensitive to the flame retardants present in the G- or F-type BcBs.

### 3.2. The Viability of Pseudomonas aeruguinosa and Bacilus subtilis in the Growth Media Containing EG or Flovan

**Figure 4:**
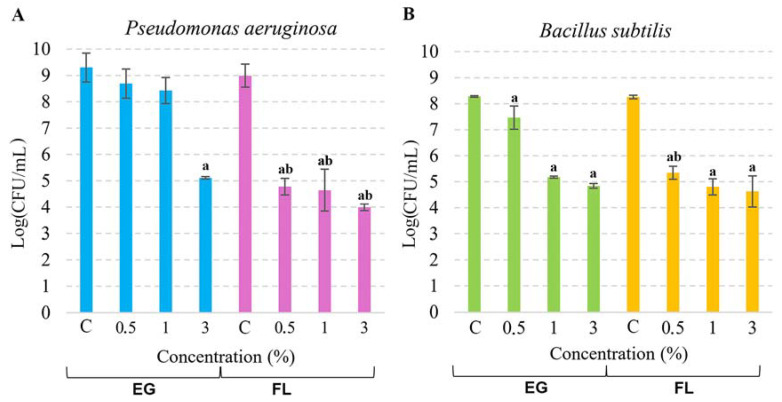
The viability of *P. aeruginosa* (**A**) and *B. subtilis* (**B**) under the action of EG or Flovan (FL). In the control experiment (depicted as “C”), bacterial cells were incubated without additives. The mean CFU/mL values were calculated, and the results are presented in the logarithmic scale. Significant deviations from the control are denoted by “a” (*p* < 0.05), while values marked by “b” are significantly different from the samples of the equivalent concentration, but different additives (*p* < 0.05).

Metagenomic analysis revealed that the H composite (without additives) had a greater variety of microorganisms, while the addition of flame retardants resulted in a decrease in microbial diversity. To analyze the antimicrobial effect of flame retardants, we were looking for bacteria which were present in both raw hemp shive samples (KS) and composites without additives (H) but missing in both additive-bearing composites (G and F). The genus-level analysis demonstrated that only two bacteria, *Bacillus* and *Pseudomonas,* satisfy these criteria (Table S1). In raw hemp shives, the source material, 0.3% of *Bacillus,* and 4.6% of *Pseudomonas* were detected. In the H-type composite, the abundance of *Bacillus* and *Pseudomonas* was reduced to 0.2% and 0.3%, respectively. However, the addition of flame retardants EG or Flovan led to an absence of this genus-level bacteria. Therefore, to further investigate the impact of flame-retardant additives on Gram-positive and Gram-negative bacteria, we selected two model microorganisms from the *Bacillus* and *Pseudomonas* genera, namely *B. subtilis* and *P. aeruginosa* (Figure 4). We used microbiological methods to evaluate the antimicrobial effects of both compounds against *B. subtilis* and *P. aeruginosa*. Incubation with EG at a concentration of 3% for 2 h resulted in a 4-log reduction in *P. aeruginosa* cells, whereas 0.5% and 1% EG samples had no significant impact. On the other hand, Flovan exhibited a more pronounced effect on *P. aeruginosa* cells, with a 5-log decrease observed in the 3% sample and a roughly 4-log cell decrease in the 0.5% and 1% samples. Gram-positive *B. subtilis* bacteria were also affected by the flame retardants, with a 3-log decrease observed in the 1% and 3% EG samples after 2 h of incubation. While the antimicrobial activity of the 0.5% EG sample was relatively low, only around a 0.5-log cell reduction was observed. The antimicrobial activity of Flovan was similar in samples with different EG concentrations—the number of cells was reduced by 3–3.5 log.

## 4. Discussion

Biocomposites combine a matrix (such as polymers or binders) with natural fibers to create a strong and eco-friendly material. Hemp shive (HS)-based composite materials are environmentally friendly and meet the High-Quality Environmental (HQE) standard, with a low total environmental effect thanks to their carbon dioxide storage capacities [34]. Hemp shives are chosen for their structural properties, including strength, stiffness, and low density, making them suitable for reinforcing the composite material and for their native antibacterial properties [35,36]. There is also a growing interest in developing flame retardants with antimicrobial activity because they can provide additional protection against microbial growth in environments where fire safety is critical. 

The presented research provides valuable data on the impact of flame-retardant additives on the bacterial composition of hemp-based biocomposite materials. This study used NGS to analyze bacterial communities in different BcBs, including those with or without flame retardants, and raw hemp shives as control samples. It also evaluated the antimicrobial effects of expandable graphite and Flovan on the bacterial species found in biocomposite materials. Specifically, we investigated the impact of these flame-retardant additives on the growth of *B. subtilis* and *P. aeruginosa* using classical microbiological methods.

NGS analysis revealed significant differences in microbial diversity among the various samples. The H-type composite, which contained only hemp shives and corn starch as a binder, exhibited the highest microbial richness and diversity. In contrast, the addition of flame-retardant additives (the F and G samples) led to a decrease in microbial diversity. The composition of bacterial communities at different taxonomic levels (phylum, order, and family) showed variations depending on the presence of flame retardants. 

In the absence of flame retardants, microbial diversity increased, with other phyla such as Bacteroidetes, Actinobacteria, and Saccharobacteria (TM7) appearing in higher abundance. This could be attributed to more exposed corn starch matrix structures. The predominant bacteria identified in hemp shives and the composites with flame-retardant additives were Proteobacteria, representing the vast majority (around 99.7%) of the microbial composition. Bacteria from the Enterobacteriaceae family predominated, which is a group exhibiting a wide range of inherent resistance capabilities including biofilm formation and resistance to chemical compounds. It was found that some representatives of this family can even degrade fire retardants [37]. 

Furthermore, this study investigated the viability response of two model microorganisms, *P. aeruginosa* and *B. subtilis*, to expandable graphite and the Flovan flame-retardant compound. *B. subtilis* and *P. aeruginosa* are two types of bacteria that can be found in the environment, such as soil and water [38,39]. *B. subtilis* is generally considered a benign organism that does not cause disease in humans, animals, or plants, but it can degrade some types of biopolymers (such as cellulose, and starch) and undergo sporulation/endospore formation [40]. *P. aeruginosa* is an opportunistic pathogen that can cause serious infections in humans, especially in those with weakened immune systems [41]. The antimicrobial effects were tested at different concentrations of these additives. Both EG and the Flovan solution demonstrated significant antimicrobial activity against the tested bacteria, with higher concentrations leading to greater reductions in bacterial cell numbers. The results of the metagenomic analysis and the microbiological tests reveal that these flame retardants have a significant antimicrobial effect against both Gram-positive and Gram-negative bacteria. 

Although the antimicrobial performance of expandable graphite has not been extensively investigated, data on the antimicrobial properties of graphene, the main structural element of EG, are available. The antimicrobial properties of graphene occur when the sharp edges of graphene nanosheets cut bacterial cell wall membranes, forming pores that lead to osmotic imbalance and, subsequently, cell death [42]. Graphite is a form of carbon that has a layered structure and itself does not have antibacterial properties, but some of its derivatives or composites do. For example, graphite carbon nitride (g-C3N4) is a photocatalytic antibacterial nanomaterial that can kill bacteria by generating reactive oxygen species under light irradiation [43]. Graphene-based materials can be further modified with metal nanoparticles, polymers, antibiotics, or enzymes to enhance their antibacterial efficiency [44]. Similarly, cell death is observed when inorganic salts of phosphorus–nitrogen or their solutions are applied [45].

Our findings provide new insights into the interactions between bacteria and biocomposites and suggest new avenues for optimizing the performance and sustainability of these materials. It could be suggested that when producing new flame retardants for BcBs, one also has to test their antimicrobial activities.

## 5. Conclusions

In summary, the findings of this study revealed that the presence of flame retardants, specifically expandable graphite and the specialized solution “Flovan CGN-01”, exerts significant influence on the microbiota compositions within specific composite materials. Control composites without flame retardants exhibited a diverse array of bacteria, consisting of Proteobacteria, Bacteroidetes, Actinobacteria, and Saccharobacteria. Meanwhile, in composites with flame retardants, the number of ASVs decreased and bacteria from Proteobacteria phylum only were observed to a high extent, thus indicating an effective inhibition of bacterial growth. Moreover, our investigation extended to demonstrate the antimicrobial effects of these flame retardants against model Gram-negative (*Pseudomonas aeruginosa*) and Gram-positive (*Bacillus subtilis*) bacteria. This dual functionality highlights the potential of flame retardants as agents not only enhancing fire safety but also combating microbial growth, as well as increasing the applicability of biocomposites. 

## Figures and Tables

**Table 1:** Evaluation of the 16S rDNA sequence data conducted for raw hemp shives (KS), biocomposite without additives (H), with EG (G), and with Flovan (F).

Sample ID	Total Reads	HQ Reads	Shannon	ASVs	Faith_pd
KS	113,788	44,218	4.2	52	2.59
G	110,043	59,534	2.1	15	3.97
F	97,329	48,342	3.3	37	1.45
H	94,245	42,038	6.3	390	40.35

## Data Availability

Data are contained within the article and supplementary materials.

## Supplementary Materials

The following are available online at https://www.mdpi.com/article/10.3390/life13122306/s1, Table S1: Bacterial taxonomy abundance count for biocomposites from phylum to genus level.
